# Traumatic Anserine Folliculosis: A Case Report With Review of Differential Diagnosis

**DOI:** 10.7759/cureus.77211

**Published:** 2025-01-10

**Authors:** Sara Al Janahi, Mariam Al Hammadi, Shaden AbdelHadi

**Affiliations:** 1 Dermatology, Sheikh Khalifa Medical City, Abu Dhabi, ARE

**Keywords:** follicular keratosis of the chin, keratotic papular lesions of the chin, pediatric dermatology, skin of color, traumatic anserine folliculosis

## Abstract

Traumatic anserine folliculosis (TAF) is a less commonly reported entity, often affecting young males and associated with trauma. Clinical recognition and dermoscopy are beneficial to diagnose this condition and avoid invasive biopsies. Topical agents, such as retinoids and vitamin D analogs, have been used with good responses; however, the condition often relapses once therapy is discontinued. We discuss this entity, review differential diagnoses, and explore management options. TAF is a benign entity that can be diagnosed based on physical examination and dermoscopy, and familiarity with this entity is crucial to avoid unnecessary invasive biopsies.

## Introduction

Traumatic anserine folliculosis (TAF) is a benign dermatologic condition, notably affecting male children and adolescents, with a predilection for skin color. TAF is characterized by grouped, skin-colored follicular papules localized to the face or neck. It is usually associated with repetitive trauma, such as prolonged friction or rubbing [1,2] but can arise without an inciting event [1,3]. TAF presents a diagnostic challenge due to its resemblance to other follicular conditions, such as closed comedonal acne, keratosis pilaris, milia en plaque, and follicular mucinosis. Topical agents, such as retinoids and vitamin D analogs, have been used with good response. However, the condition often relapses once therapy is discontinued. TAF remains underreported and often misdiagnosed. This case report highlights the clinical presentation, diagnostic considerations, and management options of TAF. We share a case of a seven-year-old boy with TAF and emphasize the utility of dermoscopy as a non-invasive diagnostic tool to differentiate this entity from its mimickers.

## Case presentation

**Figure 1:**
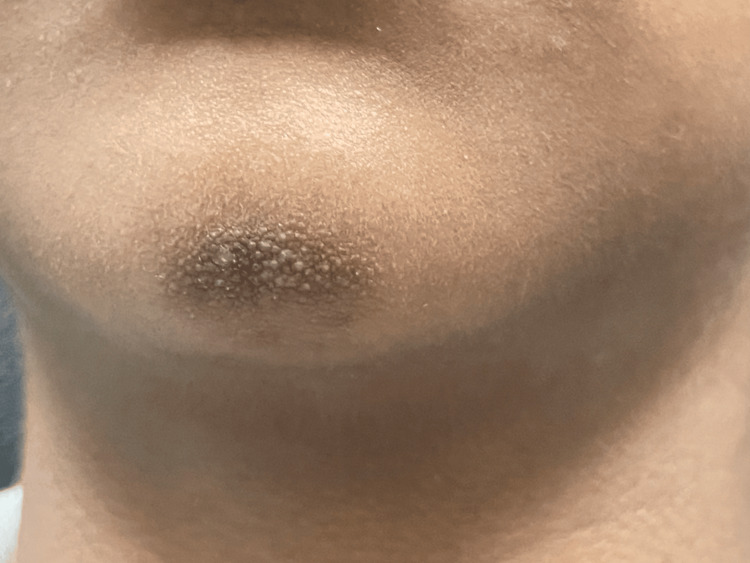
A localized plaque consisting of grouped, hyperkeratotic, skin-colored papules, overlying an ill-defined hyperpigmented patch on the chin

**Figure 2:**
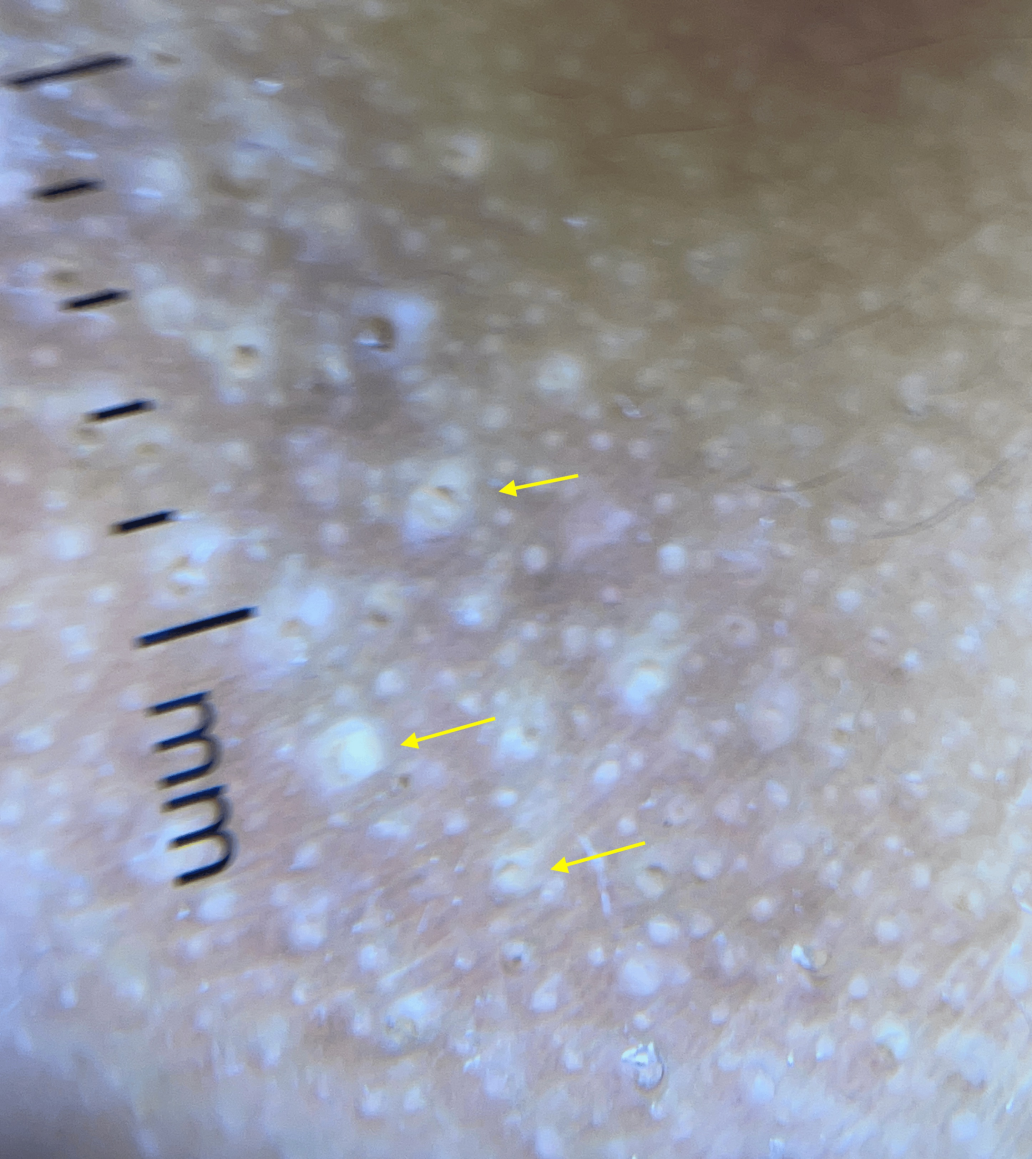
Dermoscopy revealing a series of yellow keratotic follicular plugs with spindle bodies (yellow arrows)

A seven-year-old boy with Fitzpatrick skin type (FST) V presented with a mildly pruritic plaque on the chin. The lesion had been present for a few months and failed to improve with emollients and topical steroids (hydrocortisone butyrate 0.1% cream, applied twice a day for two weeks). The patient had a background history of atopy. There was no history of preceding trauma or inflammation. The patient denied any repetitive contact with the area, such as scratching, wearing a helmet, or resting his hand on his chin for extended periods. Clinical examination revealed a localized plaque studded with a cluster of follicular hyperkeratotic skin-colored papules, overlying an ill-defined hyperpigmented patch on the chin (Figure 1). Dermoscopy revealed a group of yellow keratotic follicular plugs with spindle bodies (yellow arrows (Figure 2). Our differential diagnosis included closed comedonal acne, milia en plaque, keratosis pilaris, follicular mucinosis, and TAF. After a thorough review of the clinical presentation and dermoscopic features of each entity, a diagnosis of TAF was made.

## Discussion

TAF also termed keratotic papular lesions of the chin, or follicular keratosis of the chin (FKC) [1] is a benign entity that usually affects children and adolescents, particularly young boys. The exact pathogenesis is unclear, although the suggested etiology is trauma, specifically rubbing or resting hands on the chin [1,2]. The use of a mask during the COVID-19 pandemic was also reported as a triggering factor [4]. However, there have been reports of TAF arising without preceding trauma [1,3], as in our case. Another suggested etiological factor is the effect of androgens, considering the male predominance and the presentation in late childhood [1]. Some authors have also suggested a genetic predisposition, considering the few reported cases in siblings [3,5]. A retrospective review of 20 pediatric cases of TAF found that patients with skin color were most often affected [4], and this was also the case with our patient, who was of South Asian descent. The most commonly affected sites are the chin, jaw, or neck [6]. Presenting features are grouped, skin-colored follicular papules with a goose skin appearance (hence the term anserine, which means resembling a goose) and sandpaper-like texture [7]. Histopathology demonstrates cystic dilated hair follicles with keratotic basophilic material, staining positively with Von Kossa [3]. The presence of the stratum lucidum and a mild perivascular lymphocytic infiltrate are supporting features [7]. Dermoscopy is an appropriate diagnostic option in lieu of invasive biopsies. Observed features include dilated follicular openings with hyperkeratotic plugs containing well-demarcated yellow spindle bodies, superficial white scales [8] and milia-like cysts [1]. Additional features include fine hairs embedded in the follicle and reddish-brown structureless areas corresponding to underlying vasodilation and melanin, respectively [7]. Another non-invasive diagnostic tool is optical coherence tomography (OCT) imaging, which similarly reveals a cystic dilatation of hair follicles with keratotic plugs [9].

Differential diagnoses to consider include closed comedonal acne, keratosis pilaris, milia en plaque, and follicular mucinosis. Similarly, as in comedonal acne, comedo formation may be appreciated in TAF, likely due to an increase in sebum production and abnormal keratinization of the hair infundibulum triggered by an excess of androgens [1]. Another similar clinical feature is skin colored to whitish papules, about 1-3 mm in diameter. Histologic differentiating features include dilation of the follicular infundibulum and loose keratin and sebum in comedonal acne as opposed to dilation of the follicular orifice with TAF [7]. Follicular plugging, white scales, and a pale pink zone at the periphery sparing the surrounding skin can be seen on dermoscopy [7]. Keratosis pilaris occurs on the lateral aspects of the upper arms or anterior thighs [3], in the form of clustered 1-mm-sized, folliculocentric keratotic papules with surrounding erythema [10]. When present on the face, the lateral aspects of the cheeks are favored [3]. Dermoscopic features include perifollicular erythema, interfollicular hyperpigmentation, and coiled and twisted hair. Histopathologic features include epidermal orthokeratosis, dilated hair follicles, perivascular, and a periadnexal lymphocytic infiltrate [11]. Milia en plaque presents as multiple grouped keratinous cysts, 1-3 mm in diameter overlying an erythematous plaque. The head and neck, especially the retro-auricular and periorbital areas, are predominately involved [12]. Histopathology demonstrates multiple keratin-filled cysts, lined by a stratified squamous epithelium in the dermis. Numerous white-to-yellow cystic structures that vary in size, scattered brown dots, and telangiectatic blood vessels are seen on dermoscopy [13]. Follicular mucinosis is an important diagnosis to exclude. This entity has a similar predilection for the head and neck [14], may be idiopathic and self-limiting (often the case in children and young adults), or manifest as a feature of underlying neoplastic conditions, such as mycosis fungoides [15]. Lesions present as grouped follicular papules overlying an erythematous plaque, with non-scarring alopecia in terminal hair-bearing locations. Histopathology reveals a perifollicular mixed inflammatory infiltrate with mucin deposition within the follicular epithelium and sebaceous glands [15]. Dermoscopic features include brownish-yellow and red dots, corresponding to a dilated follicular infundibulum filled with keratotic material or sebum and dilated vessels, respectively [16].

A summary of clinical, dermoscopic, and histopathologic features is listed in Table 1.

**Table 1:** A summary of clinical, dermoscopic, and histopathologic features of traumatic anserine folliculosis and similar appearing entities

	Traumatic Anserine Folliculosis	Closed Comedonal Acne	Keratosis Pilaris	Milia en Plaque	Follicular Mucinosis
Location	Chin, jaw, neck	Face and trunk	The lateral aspects of the cheeks, upper arms or anterior thighs	Head and neck, retro-auricular and periorbital areas	Head and neck
Clinical presentation	Skin-colored grouped follicular papules with a “goose skin appearance”	Skin-colored to whitish papules of 1-3 mm in diameter	Clustered 1-mm-sized, follicular-centric keratotic papules with surrounding erythema	Multiple grouped keratinous cysts, 1-3 mm in diameter overlying an erythematous plaque	Grouped follicular papules overlying an erythematous plaque, with non-scarring alopecia in terminal hair-bearing locations
Histopathology	Dilation of the follicular infundibulum and loose keratin and sebum	Dilation of the follicular orifrice	Epidermal orthokeratosis, dilated hair follicles and perivascular and a periadnexal lymphocytic infiltrate	Histopathology demonstrates multiple keratin-filled cysts, lined by a stratified squamous epithelium in the dermis	Perifollicular mixed inflammatory infiltrate with mucin deposition within the follicular epithelium and sebaceous glands
Dermoscopy	Dilated follicular openings with hyperkeratotic plugs containing well-demarcated yellow spindle bodies, superficial white scales, milia-like cysts, fine hairs embedded in the follicle and reddish-brown structureless areas.	Follicular plugging, white scales and a pale pink zone at periphery, sparing the surrounding skin	Perifollicular erythema, interfollicular hyperpigmentation, coiled and twisted hair.	Numerous white -to-yellow cystic structures that vary in size, scattered brown dots and telangiectatic blood vessels	Brownish-yellow and red dots
References	Egawa (2021) [1], Padilha-Gonçalves (1979) [6], Arora et al. (2021) [7], Yanagihara et al. (2007) [8]	Egawa (2021) [1], Arora et al. (2021) [7]	Buechner et al. (2017) [3], Sonthalia et al. (2019) [10]	Avhad et al. (2014) [12], Chiriac et al. (2021) [13]	Alikhan et al. (2013) [14], Esteves et al. (2020) [15], Yamagishi et al. (2022) [16]

Keratolytics, such as tretinoin cream (strength unspecified) [9] and tazarotene 0.04% gel [7], have been used with good effect. Vitamin D analogs, such as tacalcitol (4.17 μg tacalcitolum monohydricum/g, Curatoderm ointment; Almirall) [3] and 0.0002% 1.24R-dihydroxyvitamin D3 (vitamin D3) ointment [8], have also been applied with complete clearance of the lesions reported in some cases [3]. A suggested mechanism for improvement with vitamin D3 analogs is a correction of epidermal differentiation and restoration of abnormal follicular keratinization [8].

Ultimately, the mainstay of treatment in trauma-induced cases is to avoid inciting traumatic behavior. A review of recent literature and TAF cases, including dermoscopic findings, is listed in Table 2.

**Table 2:** Reported cases of traumatic anserine folliculosis, with clinical and dermoscopy features and response to therapy

Reference	Clinical Presentation	History of Trauma	Dermoscopy Features	Treatment and Outcome
Egawa (2021) [1]	Siblings I: 7-year-old male with papular lesions on the chin x 6 months II: 9-year-old male with papular lesions on the chin x 3 months	No	I, II: Well-demarcated whitish yellow spindle bodies (some with a circular opening filled with a yellowish content). Also findings suggestive of a comedo formation	I: Combined topical corticosteroid and heparinoid showing mild improvement after 4 months, recurred when therapy was discontinued II: topical vitamin D3 derivative (strength unspecified)
Sil et al. (2020) [2]	10-year-old boy with asymptomatic roughness over the left cheek for 6 months	Yes - resting in a particular position, while watching television or studying	Not mentioned	Topical tretinoin cream (strength unspecified)
Buechner et al. (2017) [3]	I: 7-year-old male I: 5-year-old male siblings, both with multiple whitish, follicular, hyperkeratotic, pinpoint papules on their chins	I, II: No	I, II: Well-demarcated yellow spindle bodies	I, II: Topical tacalcitol (4.17 μg tacalcitolum monohydricum/g, Curatoderm ointment; Almirall) once daily- resulted in complete clearance in I, II, within 4 weeks. No recurrence during further 12-month follow-up
Arora et al. (2021) [7]	I: 17-year-old male with asymptomatic, raised lesions over the right cheek x 2 years II: 23-year-old male with asymptomatic raised lesions on left cheek x 1 year III: 25-year-old male with asymptomatic lesions on the left cheek x 6 months	I: history of sleeping on the right side with face in contact with the arm II: resting on the left side while watching television for hours III: resting on the left side of the face for long hours	I: dilated follicular openings, perifollicular white scales, and reddish‑brown areas with embedded hair in the follicle II: dilated follicular openings with plugs, superficial white scales, and reddish‑brown structureless areas III: dilated follicular openings with plugging	I: tazarotene 0.04% gel, with improvement after 4 weeks of therapy II: topical tretinoin cream (strength unspecified) III: topical tretinoin cream (strength unspecified)
Yanagihara et al. (2007) [8]	6-year-old male with skin-colored follicular papules, resembling gooseflesh on the chin	Yes - habitually rested his chin on the back of his hand while drawing	Well-demarcated yellow spindle bodies	Vitamin D3 ointment 2x/ day resulted in clearance however, recurrence was observed when the therapy was discontinued

## Conclusions

TAF is characterized by grouped, skin-colored follicular papules on the chin, jaw, or neck. While its exact etiology remains unclear, trauma and androgen influence are thought to play roles. Key diagnostic tools include histopathology and dermoscopy, which differentiate TAF from similar conditions such as closed comedonal acne, keratosis pilaris, milia en plaque, and follicular mucinosis. Management often involves topical treatments, such as topical retinoids or vitamin D analogs. Clinician awareness of TAF is crucial to diagnosing and managing this condition, reducing unnecessary invasive procedures, and improving patient care.

## References

[1] Egawa K (2021). Familial occurrence of follicular keratosis of the chin. J Dermatol.

[2] Sil A, Das A (2020). Traumatic anserine folliculosis. Indian Pediatr.

[3] Buechner AA, Theiler M, Krayenbuehl B, Weibel L (2018). Topical tacalcitol for family occurrence of follicular keratosis of the chin. JAMA Dermatol.

[4] Grullon K, Ashi SA, Shea CR, Ruiz de Luzuriaga AM, Stein SL, Rosenblatt AE (2022). Follicular keratosis of the face in pediatric patients of color. Pediatr Dermatol.

[5] Brenner S, Brandsen R (1992). Follicular keratosis of the chin. J Am Acad Dermatol.

[6] Padilha-Gonçalves A (1979). Traumatic anserine folliculosis. J Dermatol.

[7] Arora P, Ankad BS, Sardana K, Bansal P (2021). Traumatic anserine folliculosis and comedones: the role of dermoscopy in differentiation. Indian Dermatol Online J.

[8] Yanagihara M, Takeda K, Tanabe H, Abe S, Ishizaki H (2007). Follicular keratosis of the chin treated with 1.24R-dihydroxyvitamin D3 ointment. Pediatr Dermatol.

[9] Nguyen S, Chiaverini C, Cardot-Leccia N, Queille-Roussel C, Roussel K, Lacour JP, Bahadoran P (2016). Optical coherence tomography-assisted diagnosis of follicular keratosis of the chin. J Eur Acad Dermatol Venereol.

[10] Sonthalia S, Bhatia J, Thomas M (2019). Dermoscopy of keratosis pilaris. Indian Dermatol Online J.

[11] Gangadhar M, Adya KA, Inamadar AC (2021). A study of clinical, dermoscopic and histopathological correlation in follicular keratotic diseases: preliminary observations in 30 cases. Indian Dermatol Online J.

[12] Avhad G, Ghate S, Dhurat R (2014). Milia en plaque. Indian Dermatol Online J.

[13] Chiriac A, Wollina U, Podoleanu C, Stolnicu S (2021). Milia en plaque of the earlobe. Maedica (Bucur).

[14] Alikhan A, Griffin J, Nguyen N, Davis DM, Gibson LE (2013). Pediatric follicular mucinosis: presentation, histopathology, molecular genetics, treatment, and outcomes over an 11-year period at the Mayo Clinic. Pediatr Dermatol.

[15] Esteves M, Nogueira A, Azevedo F, Mota A (2021). Pediatric follicular mucinosis: a report of two cases. Indian Dermatol Online J.

[16] Yamagishi H, Ota M, Nobeyama Y, Asahina A (2022). Case of follicular mucinosis showing brownish yellow and red dots via dermoscopy. Clin Case Rep.

